# Nicotine Dependence and Cost-Effectiveness of Individualized Support for Smoking Cessation: Evidence from Practice at a Worksite in Japan

**DOI:** 10.1371/journal.pone.0055836

**Published:** 2013-01-30

**Authors:** Koshi Nakamura, Masaru Sakurai, Katsuyuki Miura, Yuko Morikawa, Shin-ya Nagasawa, Masao Ishizaki, Teruhiko Kido, Yuchi Naruse, Yasushi Suwazono, Hideaki Nakagawa

**Affiliations:** 1 Department of Epidemiology and Public Health, Kanazawa Medical University, Uchinada, Japan; 2 Department of Health Science, Shiga University of Medical Science, Otsu, Japan; 3 Department of Social and Environmental Medicine, Kanazawa Medical University, Uchinada, Japan; 4 School of Health Sciences, College of Medical, Pharmaceutical and Health Sciences, Kanazawa University, Kanazawa, Japan; 5 Department of Human Science and Fundamental Nursing, Toyama University School of Nursing, Toyama, Japan; 6 Department of Occupational and Environmental Medicine, Graduate School of Medicine, Chiba University, Chiba, Japan; The University of Auckland, New Zealand

## Abstract

Given the lack of economic studies evaluating the outcomes of smoking cessation programs from the viewpoint of program sponsors, we conducted a case study to provide relevant information for worksites. The present study was carried out between 2006 and 2008 at a manufacturing factory in the Toyama Prefecture of Japan and included subjects who voluntarily entered a smoking cessation program. The program included face-to-face counselling followed by weekly contact to provide encouragement over six months using e-mail or inter-office mail. Nicotine patches were available if required. All 151 participants stopped smoking immediately. Over the 24-month study period, self-report showed 49.7% abstained continuously from smoking. The rate of 24-month consecutive abstinence was higher in participants with lower Fagerström Test scores for Nicotine Dependence at baseline than in those with higher scores (63.6% for 0–2 points vs. 46.5% for 3–6 points vs. 43.8% for 7–10 points; chi-square test *p* = 0.19). A logistic regression model showed a significant linear trend for the association between the score and abstinence status after adjustment for possible confounding factors (*p* = 0.03). The crude incremental cost for one individual to successfully quit smoking due to the support program was ¥46,379 (i.e., ¥100 = $1.28, £0.83, or €1.03 at foreign exchange rates). The corresponding costs for the three categories of the Fagerström Test score for Nicotine Dependence were ¥31,953, ¥47,450 and ¥64,956, respectively. When a sensitivity analysis was conducted based on the 95% confidence interval of the success rate, the variance in the corresponding costs was ¥25,514–45,034 for 0–2 points, ¥38,344–61,824 for 3–6 points, and ¥45,698–108,260 for 7–10 points. The degree of nicotine dependence may therefore be an important determinant of the cost-effectiveness of smoking cessation programs.

## Introduction

Cigarette smoking is a crucial but avoidable cause of premature disability and death due to cardiovascular disease, cancer, and respiratory tract disease [1]–[3]. In Japan, where the rate of smoking in males exceeds that of males in developed Western countries [4], [5], 38.6% of cancer-related deaths, 23.4% of deaths due to respiratory tract disease, and 23.0% of deaths due to cardiovascular disease among Japanese males have been attributed to a history of smoking [2]. Smoking is burdensome because of the effects on premature disability and excess mortality [2], [3] and also because of the substantial costs to medical insurers in Japan [6], [7]. Izumi et al [6] showed that over a follow-up period of 30 months, Japanese males with a history of smoking incurred medical costs averaging ¥3,000 per month in excess of the costs attributed to individuals who had never smoked. As a result, up to four percent of total medical expenditures in the Japanese population may be attributable to smoking [6].

A key strategy for promoting smoking cessation in people intending to quit is to provide individualized support including professional counselling and pharmacological therapy [8], [9]. In addition to assistance rendered in clinics and hospitals, employers can also offer similar support given that health care providers, such as occupational health physicians and nurses, are available at worksites [10]–[15]. Furthermore, worksites present favorable settings for implementing smoking cessation programs for several reasons. First, worksites employ young adult and middle-aged smokers who are candidates for early smoking cessation intervention. Second, the work environment readily promotes favorable interaction between employees attempting to stop smoking and health care providers, as they are peers who all belong to the same worksite. Third, easy accessibility to both health care providers and employees in the worksite is advantageous as it allows flexible delivery of smoking cessation therapies without the need for missing work or having communications in private. Finally, all employers are responsible for managing the health care of their employees.

Previous studies, including randomized controlled trials and case studies, have examined the effectiveness of various worksite support systems for smoking cessation in Japan [10]–[15] and in other countries [16]–[22]. However, in the majority of previous studies the follow-up periods were incomplete and the status of successful smoking cessation was assessed for only 12 months after program initiation despite evidence that some individuals may resume smoking even after abstaining for one year [23], [24]. Therefore, the outcomes of individuals attempting to quit smoking may be inaccurately reflected in the current published literature. Furthermore, in spite of the practical importance of considering both the effectiveness and cost-effectiveness of smoking cessation programs, economic evidence supporting the implementation of these programs, particularly from the perspective of program sponsors, is limited throughout the world [16], [25]–[28]. To assist with promoting smoking cessation in the worksite, we conducted a case study with the aim of providing preliminary information on the effectiveness and cost-effectiveness of an individualized support program at a worksite in Japan. First, we employed a two-year follow-up period in order to evaluate the long-term outcomes of individuals who participated voluntarily in a six-month program at a single worksite. Second, we estimated the cost-effectiveness of this program from the perspective of the employer who paid for this program. Finally, we evaluated these outcomes in the subjects stratified by nicotine dependence status. Published studies have indicated that compared to smokers who scored lower on measures of nicotine dependence, smokers who scored higher have more frequently elected to use nicotine replacement therapy [29] but have succeeded in smoking cessation less often [30]. The guideline also recommends physicians to consider nicotine replacement therapy more positively in the case of greater nicotine dependence [31]. Nicotine dependence, therefore, may influence the cost-effectiveness of smoking cessation programs.

## Methods

### Study setting and participants

This case study was conducted at a metal products factory in the Toyama prefecture of Japan. Approximately 4,800 male and 2,500 female workers were employed at this factory, where smoking is allowed in limited areas. At the initiation of the study in 2006, an annual health examination conducted in the same year indicated that 48.4% of male workers and 6.1% of female workers, aged 20 years or older were active smokers. During the three-year interval spanning the fiscal years 2006 and 2008, a total of 155 workers who smoked (150 men and five women) participated voluntarily in the study and signed a smoking cessation declaration form. We allocated all the participants to the intervention group without establishing a control group. The study was approved by the Institutional Review Committee of Kanazawa Medical University for Ethical Issues.

### Smoking cessation program

A worksite clinic at the target factory employed an occupational health physician and six nurses with each of the nurses sharing responsibility for the participants. Using an individualized approach, the smoking cessation program consisted of the following three steps. First, in order to educate the participants on the hazards of smoking and the benefits of cessation and to strengthen self-efficacy, the occupational health physician and a nurse conducted a joint introductory face-to-face counselling session at the worksite clinic. At this time, the participants provided information on the number of cigarettes smoked each day, cessation history, nicotine dependence, alcohol drinking habits, and disease profiles through self-reported questionnaires or physical and medical records of annual health examinations. The levels of nicotine dependence were assessed using the Fagerström Test for Nicotine Dependence (FTND) [32]. The participants were next required to submit a diary on smoking cessation via e-mail or inter-office mail once a week for a period of six months. The responsible nurse inspected the diary to confirm smoking cessation status and then provided a tailor-made, encouraging comment every week. For participants who did not submit a diary, the nurse confirmed smoking cessation status every month. Lastly, nicotine replacement therapy was offered to participants who desired to use it or if the physician considered this therapy would be beneficial after reference to the FTND scores and/or the number of cigarettes smoked each day. The nicotine patches were the only aid used to simplify the study design. The physician basically followed the Manual on Smoking Cessation Support issued by relevant Japanese medical societies, which recommends that nicotine replacement therapy is more applicable to smokers with greater nicotine dependence, starting with 30 mg patches [31]. However, the study left this matter to the discretion of the physician and/or desire of the participant, as is often the case in a practice setting, a fact that this study regarded as important. Participants who elected nicotine replacement therapy paid for 30% of the treatment costs. Only nicotine patch users were required to visit the worksite clinic mainly to obtain a prescription for this medication. This support was stopped at the end of the sixth month. Incentives were also offered, with participants who abstained for six consecutive months being congratulated for completing the six-month support program, and presented with an award. Finally, smoking cessation status was assessed on the basis of self-reporting after a further 18 months of follow-up, during which time no support was provided. Individuals who refrained from smoking for 24 consecutive months after initially stopping were regarded as successful quitters.

### Other smokers at the factory

We also examined the characteristics of workers who smoked and did not participate in the smoking cessation support program. In addition, we examined the smoking pattern in these non-participating smokers over a two-year follow-up period from 2006 to 2008 in order to estimate the natural quit rate in smokers at the target factory. In employees who underwent an annual health examination in 2006 (enrolment rate >90% of total employees), there were 2,216 non-participating smokers, aged 20 years or older (2,069 men and 147 women), after excluding the 155 smokers who received support to stop smoking. Data were collected from physical and medical records obtained at the annual health examinations on smoking habits, cigarettes smoked each day, alcohol drinking habits, disease profiles in 2006, and smoking habits in 2008. This procedure was conducted as part of a cohort study that consisted of workers in the factory who provided their approval to take part after considering the ethical issues. The details of the study subjects and methods have been described elsewhere [33], [34].

### Data analysis

First, we calculated the rate of consecutive abstinence in the 24th month after cessation of smoking had commenced. Next, using reference to a previous study [25], we assessed the cost-effectiveness of our support program from the viewpoint of the employer who was the program sponsor. In this economic evaluation, the costs were expressed in Japanese Yen, with the program costs consisting of the following three components: 1) material costs (diaries, documents, nicotine patches, and awards), 2) opportunity costs for health care providers, and 3) opportunity costs for participants, which represented the loss in production time due to attendance at the worksite clinic for initial counselling, and prescription of nicotine patches and diary entries during work time. To calculate the opportunity costs for participants, this study assumed that the employer who was the program sponsor allowed the participants to engage in these activities during work time. No developmental costs were incurred based on the assumption that the program did not require the health care providers to have specialized skills for smoking cessation. The opportunity costs for health care providers were calculated by multiplying the time (hours) spent for each procedure by the respective average 2006 hourly salary rates (¥/hour) for physicians and workers at manufacturing companies in Japan [35]. The crude incremental cost for one individual to successfully quit smoking due to the support program was calculated as the total costs incurred in the program divided by the number of 24-month consecutive abstainers. Because success rate is an important determinant of cost-effectiveness [36], we performed a sensitivity analysis to evaluate the extent to which the cost-effectiveness changed in response to variations in success rate. The 95% confidence interval of success rate was calculated for test parameters used in the sensitivity analysis. The upper limit of the 95% confidence interval was calculated using F-distribution functions as: {2(y+1)×F_(2(y+1), 2(n−y); 0.05/2)_}/{2(y+1)×F_(2(y+1), 2(n−y); 0.05/2)_+2(n−y)}, where F_(2(y+1), 2(n−y); 0.05/2)_ was the F-value with 2(y+1) and 2(n−y) degrees of freedom, n was the number of participants, and y was the number of successful quitters [37]. The lower limit of the 95% confidence interval was calculated as: 2y/{2(n−y+1)×F_(2(n−y+1), 2y; 0.05/2)_+2y}, where F_(2(n−y+1), 2y; 0.05/2)_ was the F-value with 2(n−y+1) and 2y degrees of freedom, and n and y were the same as described above [37]. Variance in the number of successful quitters was determined by multiplying the number of participants by the lower and upper limits of the success rate. The lower and upper limits of incremental cost for one individual to successfully quit smoking due to the support program was then calculated as the total costs incurred in the program divided by the upper and lower limits of the number of successful quitters, respectively.

It is likely that some of our study participants may have successfully stopped smoking without our support (i.e., natural quitters) [13], [25]. It is therefore necessary to evaluate the net cost-effectiveness, taking into account individuals who stopped smoking unassisted. Using the results on the natural quit rate in smokers who had not received support to stop smoking, we calculated the number of abstainers in an unassisted situation as the number of participants multiplied by the natural quit rate. The net incremental cost for one individual to successfully quit smoking due to the support program was calculated as {(total costs incurred in the program) minus (no costs incurred in an unassisted situation)} divided by {(the number of 24-month consecutive abstainers in the support program) minus (the number of abstainers in an unassisted situation)} [38].

Finally, the chi-square test was used to compare the rates of 24-month consecutive abstinence in participants grouped according to the FTND score at baseline (0–2 points, 3–6 points or 7–10 points, classified as mild, moderate, or severe nicotine dependence, respectively [32]). The significance of the linear trend for the association between FTND score (continuous variable) and successful smoking cessation was tested using a logistic regression model that incorporated the following variables as covariates: age (continuous variable), sex (male or female), cigarettes smoked each day (continuous variable), cessation history (yes or no), alcohol drinking habits (drinker or non-drinker (including occasional drinker)), and history of either heart disease, stroke, cancer, chronic respiratory disease, hypertension (defined as a systolic blood pressure ≥140 mmHg, diastolic blood pressure ≥90 mm Hg and/or taking medication for hypertension), hypercholesterolemia (defined as a serum low-density lipoprotein cholesterol ≥3.62 mmol/l (140 mg/dl) and/or taking medication for hypercholesterolemia), or diabetes (defined as a the Japan Diabetes Society-Hb_A1c_≥6.1 % (or the National Glycohemoglobin Standardization Program-Hb_A1c_≥6.5 %) and/or taking medication for diabetes). Following stratification of the participants into the three levels of nicotine dependence, defined by the baseline FTND scores, we assessed the effect of varying the rates of nicotine patch use and smoking cessation success on cost-effectiveness. It has been reported that these factors may be influenced markedly by FTND score [29]–[31]. In addition, we performed a similar sensitivity analysis using the various FTND scores, basing the test parameters for the sensitivity analysis on the 95% confidence interval of success rate which was calculated in a similar manner.

The statistical analyses were performed using the Statistical Package for the Social Sciences Version 12.0J for Windows (SPSS Japan Inc., Tokyo, Japan). All probability values were two-tailed and the significance level was set at *p*<0.05.

## Results

### Characteristics of study participants

Of the 155 smokers who participated voluntarily in the study (150 men and five women), one participant retired one month after stopping smoking without relapse and further three participants had missing baseline survey data. After excluding these four participants, the remaining 151 participants (146 men and five women) were considered eligible for inclusion in the analysis.

At baseline, the mean age±standard deviation of the 151 study participants was 44.2±11.2 years (Table 1). The mean number of cigarettes smoked each day was 20.5±7.5, while the mean FTND score was 4.6±2.5 points. The FTND scores of the participants were distributed as follows: 21.9% scored 0–2 points, 57.0% scored 3–6 points, and 21.2% had 7–10 points. Of the participants, 46.4% had attempted to quit smoking previously, and 55.6% had a health problem at study entry (defined as any combination of a history of heart disease, stroke, cancer, chronic respiratory disease, hypertension, hypercholesterolemia, and/or diabetes), while 3.3% had a severe health problem (defined as any combination of heart disease, stroke, cancer, and/or chronic respiratory disease). When stratified according to the FTND score, the means of age and cigarettes smoked each day and the prevalence of having previously attempted to quit smoking and having a severe health problem were higher with increasing FTND score.

**Table 1:** Baseline characteristics of the 151 smokers who participated in the support program at a worksite in Toyama, Japan, and the 2,166 smokers who did not participate in the study.

	Participating smokers				Non-	*p* value	*p* value	*p* value
	Overall	Fagerström Test score for Nicotine Dependence			participating	for	for	for
	(n = 151)	0–2 points	3–6 points	7–10 points	smokers	difference^a^	difference^b^	linear trend^c^
		(mild)	(moderate)	(severe)	(n = 2,166)			
		(n = 33)	(n = 86)	(n = 32)				
Age, yrs	44.2±11.2	40.4±12.5	44.5±11.3	47.2±8.3	42.5±11.3	0.08	0.05	<0.01
Female, % (n)	3.3 (5)	6.1 (2)	3.5 (3)	0 (0)	6.0 (130)	0.17	0.39	0.19
Cigarettes smoked each day, n	20.5±7.5	14.1±6.3	20.5±5.1	27.2±8.2	18.2±7.4	<0.01	<0.01	<0.01
≤10 per day, % (n)	13.9 (21)	42.4 (14)	8.1 (7)	0 (0)	22.5 (487)			
11–20 per day, % (n)	60.9 (92)	57.6 (19)	69.8 (60)	40.6 (13)	60.7 (1,314)			
21–30 per day, % (n)	20.5 (31)	0 (0)	22.1 (19)	37.5 (12)	14.6 (316)			
≥31 per day, % (n)	4.6 (7)	0 (0)	0 (0)	21.9 (7)	2.3 (49)			
Fagerström Test score for								
Nicotine Dependence, point	4.6±2.5	1.2±0.9	4.6±1.1	8.0±1.0	Not available			
History of attempt to quit smoking, % (n)	46.4 (70)	69.7 (23)	40.7 (35)	37.5 (12)	Not available		<0.01	<0.01
Habitual alcohol drinking, % (n)	75.5 (114)	75.8 (25)	76.7 (66)	71.9 (23)	72.4 (1,569)	0.42	0.86	0.21
History of heart disease, % (n)	0.7 (1)	0 (0)	0 (0)	3.1 (1)	1.4 (31)	0.43	0.15	0.98
History of stroke, % (n)	0 (0)	0 (0)	0 (0)	0 (0)	0.3 (6)	0.52		
History of cancer, % (n)	2.0 (3)	0 (0)	1.2 (1)	6.3 (2)	0 (0)	<0.01	0.13	0.18
History of chronic respiratory disease, % (n)	0.7 (1)	0 (0)	1.2 (1)	0 (0)	0.05 (1)	0.01	0.68	0.61
History of hypertension^d^, % (n)	25.2 (38)	18.2 (6)	26.7 (23)	28.1 (9)	20.2 (438)	0.15	0.57	0.73
History of hypercholesterolemiad, % (n)	35.1 (53)	30.3 (10)	32.6 (28)	46.9 (15)	33.3 (721)	0.65	0.28	0.30
History of diabetes^d^, % (n)	10.6 (16)	6.1 (2)	11.6 (10)	12.5 (4)	5.4 (117)	<0.01	0.63	0.98
History of a health problem^e^, % (n)	55.6 (84)	45.5 (15)	54.7 (47)	68.8 (22)	48.1 (1,042)	0.07	0.16	0.46
History of a severe health problem^e^, % (n)	3.3 (5)	0 (0)	2.3 (2)	9.4 (3)	1.8 (38)	0.17	0.08	0.06

The data are presented for all the participating smokers and also grouped according to the Fagerström Test score for Nicotine Dependence at baseline. Values are expressed as mean ± standard deviation, or the % (number) of individuals in that category. a. An unpaired t test or a chi-square test was used to compare each factor between the participating and non-participating smokers. b. One-way analysis of variance, or a chi-square test was used to compare each factor among the three categories of the Fagerström Test score for Nicotine Dependence. c. A logistic regression model was used to test the significance of the linear trend for the association between Fagerström Test score for Nicotine Dependence (continuous variable) and each factor after adjustment for age. d. Hypertension was defined as a systolic blood pressure≥140 mmHg, diastolic blood pressure≥90 mmHg and/or taking medication for hypertension; hypercholesterolemia as a serum low-density lipoprotein cholesterol ≥3.62 mmol/l and/or taking medication for hypercholesterolemia; diabetes as a Japan Diabetes Society-Hb_A1c_≥6.1 % (or the National Glycohemoglobin Standardization Program-Hb_A1c_≥6.5 %) and/or taking medication for diabetes. e. A health problem was defined as any combination of the seven diseases listed above; a severe health problem as any combination of heart disease, stroke, cancer and/or chronic respiratory disease.

The means of age and cigarettes smoked each day and the prevalence of having a health problem were higher in smokers who participated in the smoking cessation support program than in the 2,166 non-participating smokers, after exclusion of those with missing baseline survey data (n = 50).

### Success rate in the study participants

Nicotine patches were used by 61.6% (n = 93) of all the participants, with the total quantities consumed by the study population being 1,091 person-pieces for 30 mg patches, 889 person-pieces for 20 mg patches, and 560 person-pieces for 10 mg patches (Table 2). Nicotine patches were used more frequently by participants with higher baseline FTND scores compared to those with lower baseline scores: 30.3% (n = 10) for participants with a FTND score of 0–2 points, 62.8% (n = 54) for those with a score of 3–6 points, and 90.6% (n = 29) for those with a score of 7–10 points. Quantities consumed per user were also higher with increasing baseline FTND score: 15.4 pieces for FTND 0–2 points (30 mg, 4.1 pieces; 20 mg, 7.7 pieces; and 10 mg, 3.6 pieces), 24.7 pieces for FTND 3–6 points (30 mg, 9.9 pieces; 20 mg, 9.1 pieces; and 10 mg, 5.7 pieces), and 36.3 pieces for FTND 7–10 points (30 mg, 17.8 pieces; 20 mg, 11.1 pieces; and 10 mg, 7.4 pieces). Of the participants using nicotine patches, the 30 mg patches were used by 40.0% (n = 4) for 0–2 points, 77.8% (n = 42) for 3–6 points and 93.1% (n = 27) for 7–10 points. No participants received any other relevant support for smoking cessation, or used any other relevant medication such as nicotine gum during the follow-up period.

**Table 2:** Costs (Japanese Yen) of the support program for smoking cessation at a worksite in Toyama, Japan.

			Details	Overall	Fagerström Test score for Nicotine Dependence		
				(n = 151)	0–2 points	3–6 points	7–10 points
					(mild)	(moderate)	(severe)
					(n = 33)	(n = 86)	(n = 32)
Material costs							
	Diaries and documents		¥300×151 people (33, 86 and 32)	¥45,300	¥9,900	¥25,800	¥9,600
	Nicotine patches						
		30 mg	¥278×1,091 person-pieces (41, 533 and 517)	¥303,298	¥11,398	¥148,174	¥143,726
		20 mg	¥262×889 person-pieces (77, 491 and 321)	¥232,918	¥20,174	¥128,642	¥84,102
		10 mg	¥248×560 person-pieces (36, 308 and 216)	¥138,880	¥8,928	¥76,384	¥53,568
	Awards		¥5,000×88 people (22, 48 and 18)	¥440,000	¥110,000	¥240,000	¥90,000
Opportunity costs for physician							
	Initial counselling		¥4,640×0.1 hours×151 people (33, 86 and 32)	¥70,064	¥15,312	¥39,904	¥14,848
	Prescription						
		of nicotine patches	¥4,640×0.05 hours×201 person-times (4, 107 and 90)	¥46,632	¥928	¥24,824	¥20,880
Opportunity costs for nurse							
	Initial counselling		¥1,800×0.3 hours×151 people (33, 86 and 32)	¥81,540	¥17,820	¥46,440	¥17,280
	Prescription						
		of nicotine patches	¥1,800×0.05 hours×201 person-times (4, 107 and 90)	¥18,090	¥360	¥9,630	¥8,100
	Checking diaries						
		and providing comments	¥1,800×0.1 hours×2,797 person-times (665, 1,538 and 594)	¥503,460	¥119,700	¥276,840	¥106,920
	Conferring an award						
		to 6-month abstainers	¥1,800×0.1 hours×88 people (22, 48 and 18)	¥15,840	¥3,960	¥8,640	¥3,240
	Other routine work		¥1,800×0.3 hours×151 people (33, 86 and 32)	¥81,540	¥17,820	¥46,440	¥17,280
Opportunity costs for participants							
	Initial counselling^a^		¥1,800×(0.3+0.2) hours×151 people (33, 86 and 32)	¥135,900	¥29,700	¥77,400	¥28,800
	Prescription						
		of nicotine patches^a^	¥1,800×(0.05+0.2) hours×201 person-times (4, 107 and 90)	¥90,450	¥1,800	¥48,150	¥40,500
	Keeping a diary		¥1,800×0.05 hours×13,985 person-times (3,325, 7,690 and 2,970)	¥1,258,650	¥299,250	¥692,100	¥267,300
	Being presented with an award		¥1,800×0.1 hours×88 people (22, 48 and 18)	¥15,840	¥3,960	¥8,640	¥3,240
Total				¥3,478,402	¥671,010	¥1,898,008	¥909,384

The data are presented for all the participating smokers and also grouped according to the Fagerström Test score for Nicotine Dependence at baseline. ¥100 = $1.28, £0.83, or €1.03 at the foreign exchange rates on June 1, 2012. Values in parentheses represent the respective values in participants who had 0–2, 3–6, and 7–10 points for the Fagerström Test score for Nicotine Dependence. a. Participants spent an additional 0.2 hours during work time for the initial counselling and prescription of the nicotine patches, due to the need to commute between the work place and the clinic.

**Figure 1:**
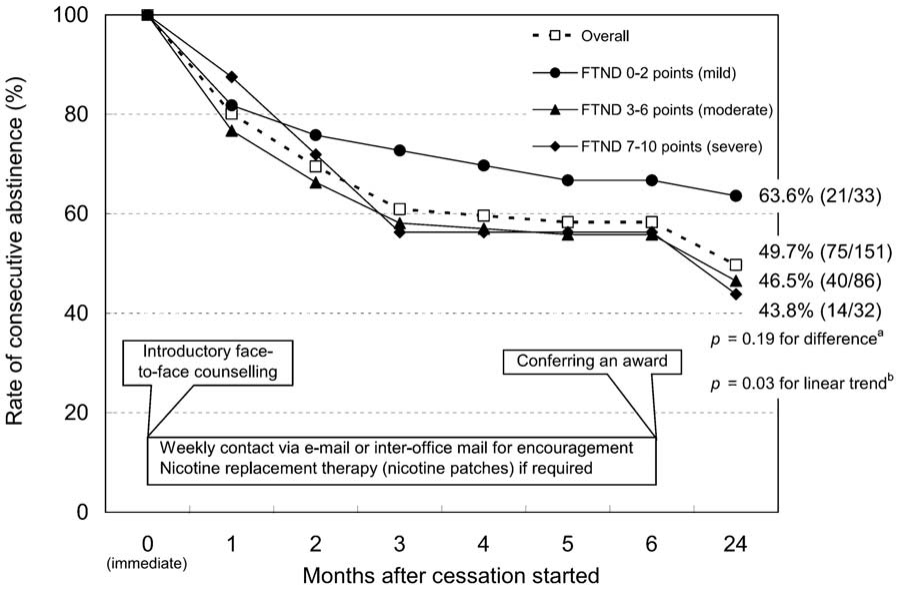
Scheme of the six-month support program and time-related trend in the rate of consecutive abstinence in the 151 study participants at a worksite in Toyama, Japan, after the start of smoking cessation. Data are presented for the entire study population and also grouped according to the Fagerström Test for Nicotine Dependence (FTND) score at baseline. A chi-square test (a) was used to compare the rate of 24-month consecutive abstinence among the three categories of FTND score, while a logistic regression model (b) was used to test the significance of the linear trend for the association between FTND score (continuous variable) and 24-month consecutive abstinence after adjustment for age, sex, cigarettes smoked each day, cessation history, alcohol drinking habits, and history of either heart disease, stroke, cancer, chronic respiratory disease, hypertension, hypercholesterolemia, or diabetes. Values in parentheses represent the number of successful quitters/study participants.

The rate of consecutive abstinence in the 151 study participants decreased gradually in the months following the initial cessation of smoking (Figure 1). Over the 24 study months, 49.7% refrained continuously from smoking. Participants with lower baseline FTND scores tended to be successful quitters more frequently compared to those with higher baseline scores (63.6% for 0–2 points vs. 46.5% for 3–6 points vs. 43.8% for 7–10 points). Although the difference in the rates of successful cessation among the three categories was not statistically significant (*p* = 0.19), the test for linear trend reached statistical significance (*p* = 0.03). This indicated that the odds ratio for successful quitting associated with a one point increase in FTND score was 0.81 (95% confidence interval, 0.66–0.98) after adjustment for age, sex, cigarettes smoked each day, cessation history, alcohol drinking habit, and history of each of the seven diseases listed.

### Natural quit rate in the non-participating smokers

Of the 2,166 non-participating smokers, 2,049 (1,920 men and 129 women) aged 20–58 years were included in the cohorts used to estimate the natural quit rate, and were followed-up for two years from 2006 to 2008. Smokers aged 59–60 years were excluded, as they were required to retire by the end of this follow-up period. Of the 2,049 subjects in the cohort, 1,764 (1,651 men and 113 women) provided information on smoking status at an annual health examination in 2008, and were considered eligible for inclusion in the data analysis.

Over the two-year follow-up, 173 individuals ceased smoking. Therefore, the point-prevalence of abstinence in the non-participating smokers was 9.8% (data not shown in the table). The rate of 24-month consecutive abstinence in the smokers who participated in the cessation program was significantly higher than this value (*p*<0.01).

### Economic evaluation for this support for smoking cessation

The support program incurred an estimated total cost of ¥3,478,402 (Table 2 and 3). Material costs, opportunity costs for health care providers (physician and nurse), and opportunity costs for participants accounted for 33.4%, 23.5% (3.4% for physician and 20.1% for nursing costs), and 43.1% of total expenditures, respectively. The crude incremental cost for one individual to successfully quit smoking due to the support program was ¥46,379 (i.e., ¥100 = $1.28, £0.83, or €1.03 at the foreign exchange rate on June 1, 2012). The corresponding costs varied across the three categories of FTND scores: ¥31,953 for 0–2 points, ¥47,450 for 3–6 points, and ¥64,956 for 7–10 points. Material costs accounted for a larger proportion of total costs in participants with higher FTND scores, compared with those with lower FTND scores. The sensitivity analysis demonstrated that varying the smoking cessation success rate across a range of values defined by the 95% confidence interval resulted in alterations in the cost-effectiveness of the support program.

**Table 3:** Incremental costs (Japanese Yen) for one individual to successfully quit smoking due to the support program at a worksite in Toyama, Japan.

			Overall	Fagerström Test score for Nicotine Dependence		
				0–2 points	3–6 points	7–10 points
				(mild)	(moderate)	(severe)
Participants, n 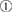			151	33	86	32
24-month consecutive abstinence rate, % 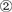			49.7	63.6	46.5	43.8
		Lower limit of 95% confidence interval	41.4	45.1	35.7	26.4
		Upper limit of 95% confidence interval	57.9	79.6	57.6	62.3
24-month consecutive abstainers, n 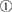 × 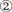 /100 ( = 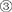 )			75	21	40	14
		Lower limit	62.5	14.9	30.7	8.4
		Upper limit	87.4	26.3	49.5	19.9
Total costs of support program, ¥ 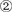			¥3,478,402	¥671,010	¥1,898,008	¥909,384
	Material costs, % of total		33.4	23.9	32.6	41.9
	Opportunity costs for physician, % of total		3.4	2.4	3.4	3.9
	Opportunity costs for nurse, % of total		20.1	23.8	20.4	16.8
	Opportunity costs for participants, % of total		43.1	49.9	43.5	37.4
Crude incremental costs for one individual to successfully quit smokinga,						
	¥ 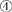 / 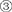		¥46,379	¥31,953	¥47,450	¥64,956
		Lower limit	¥39,799	¥25,514	¥38,344	¥45,698
		Upper limit	¥55,654	¥45,034	¥61,824	¥108,260
Net incremental costs for one individual to successfully quit smokinga,						
	¥ ( 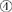 -0)/{ 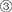 -( 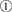 ×9.8/100^b^)}		¥57,781	¥37,697	¥60,064	¥83,430
		Lower limit	¥47,912	¥29,048	¥46,180	¥54,130
		Upper limit	¥72,922	¥57,351	¥85,112	¥171,582

The data are presented for all the participating smokers and also grouped according to the Fagerström Test score for Nicotine Dependence at baseline. ¥100 = $1.28, £0.83, or €1.03 at the foreign exchange rates on June 1, 2012. a A sensitivity analysis of the incremental costs for one individual to successfully quit smoking was conducted based on the 95% confidence interval of the success rate. b Natural quit rate (9.8%) was estimated on the basis of the two-year point-prevalence of abstinence in the 1,764 smokers who did not participate in the program.

Given that 9.8% of the participants succeeded in stopping smoking unassisted, the net incremental cost for one individual to successfully quit smoking due to the support program was ¥57,781. There was a variation of ¥37,697-83,430, when this calculation was conducted according to the FTND score.

## Discussion

One merit of our study is that it achieved almost complete, long-term follow-up of individuals who had voluntarily attempted to stop smoking and had received individualized support at a worksite in Japan. In a study of 166 subjects who underwent individualized support for smoking cessation at a worksite in Japan, Takayama [14] found that the rate of self-reported abstinence from smoking was 59.6% after 12 months, whereas Sawayama et al. [15] reported corresponding rates of 48.5% after 12 months in 66 subjects at another worksite in Japan. Previous studies in other countries also provided similar or other kinds of smoking cessation support programs for smokers at worksites (e.g., counselling by professional psychologists or former smokers, phone, television, group treatment, competition, or incentives), some of which resulted in 40–50% success rates over one-year or longer [16]–[22]. Thus, the individual support provided in our program generated outcomes that may be at least comparable to those resulting from the assistance provided in these earlier worksite-based studies. In contrast, a Japanese nationwide clinic/hospital-based survey conducted in 2007 showed that 40.8% and 32.6% of individuals who attempted to stop smoking remained self-reported abstinent after six and 12 months, respectively [39]. In this survey, the subjects received standard individual support including face-to-face counselling and nicotine replacement therapy (up to five times over 12 weeks) under the medical insurance system in Japan [31], with only nicotine patches and gums being available at that time. Varenicline is a more effective oral medication for smoking cessation than nicotine patches [9], [30], and although unavailable at the time of the study, it is now used widely in Japan.

The support provided in our program was characterized by frequent and long-term contact with the individuals attempting to stop smoking using e-mail or inter-office mail. Frequent and long-term visits to clinics or hospitals may be difficult for employees who are unwilling to be absent from work, despite evidence that this type of interaction increases the success rate [39]. In addition, e-mail and inter-office mail are readily available for everyone at worksites without charge, whereas some individuals do not have a computer at their home. Our program also offered an incentive by rewarding individuals who successfully quit smoking for six months. However, attention should be paid to several different characteristics other than the uniqueness of our worksite-based study when comparing its effectiveness with other clinic or hospital-based studies. Our study participants were relatively healthy workers, with the prevalence of a history of smoking-related serious diseases such as heart disease, stroke, cancer, or chronic respiratory disease [1]–[3] being lower than in smokers in a hospital-based survey (≤2% vs. 7–12%) [30]. In particular, our study group appeared to be well-motivated to stop smoking as evidenced by their voluntary rather than mandatory participation in the program. In contrast, at clinics or hospitals, unmotivated smokers who merely followed the advice of their physicians may also have attempted to quit smoking [30], ultimately leading to a decrease in success rate [13], [30]. In addition, many subjects attempting to quit smoking at clinics or hospitals may have various diseases [30], such as mental disorders, which may lead to difficulty in cessation [30]. Unfortunately, we have no data on mental health in our study participants. Furthermore, subjects attempting to quit smoking at clinics or hospitals may have higher FTND scores indicating greater nicotine dependence than those at our worksite [30]. Taniguchi et al. [30] reported that smokers with FTND scores ≥6 had a lower rate of four-week consecutive abstinence compared to those with FTND scores ≤5 (39.7% vs. 51.8%). This finding is in agreement with the results of our study on the association between FTND score and successful smoking cessation.

The majority of relevant economic evaluations have been reported from the United States [16], [26]–[28]. Ringen et al. [26] studied blue-collar smokers who participated voluntarily in a practical program that provided one- or five-time phone-call counselling combined with various kinds of medications. In that study, the ≥12-month point-prevalence of smoking cessation was 27.5%, and the crude incremental cost per one additional quitter was $1,025 (equivalent to approximately ¥80,000 at current exchange rates). Jason et al. [27] estimated that cost per one additional quitter (12-month point prevalence to continuous abstinence) was $225–1,179 (approximately ¥17,600–92,100) for self-help, $250–699 (approximately ¥19,500–54,600) for incentives, and $455–790 (approximately ¥35,500–61,700) for group treatment. Similarly, Ong et al. [28] estimated that the cost per 12-month quitter was $7,020 (approximately ¥548,400) for nicotine replacement therapy using patches and gums and $799 (approximately ¥62,400) for a smoke-free workplace policy. In Japan, the High-Risk and Population Strategy for Occupational Health Promotion Study (HIPOP-OHP) [25], that conducted a smoking cessation intervention trial at manufacturing factories in the early 2000 s, reported that factories providing intervention had a higher point-prevalence of smoking cessation after 36 months of follow-up in all workers who smoked compared to control factories (12.1% vs. 9.4%). That trial employed a mandatory, low intensity, comprehensive intervention for all smokers that consisted of the following four components: (1) presenting information on the harms of smoking and the benefits of cessation using posters and other relevant materials, (2) a smoking cessation campaign, (3) advice on the designation of smoking areas, and (4) periodic site visits of the designated smoking areas. The net incremental cost for one individual to successfully quit smoking due to this comprehensive intervention was ¥70,080, compared to no costs without intervention [25]. Whereas the calculated costs included developmental costs, the net incremental cost was reduced to approximately ¥60,000 when developmental costs were omitted from the cost-effectiveness analysis [25]. Although we were unable to reach a definite conclusion due to our small sample size and heterogeneous methods for calculating costs, targeting our intensive intervention to individual smokers who showed a willingness to quit may be comparable to the majority of interventions used in these earlier studies. Importantly, our data suggest it is relatively less cost-effective to promote smoking cessation in smokers who are willing to quit but are severely dependent on nicotine compared to those who are mildly dependent. This is due mainly to a higher rate and greater quantity of nicotine patch use and a lower rate of successful smoking cessation in the former group. It is therefore necessary to pay attention to factors related to nicotine dependence (e.g., nicotine dependence status of study participants, and costs of this therapy) when evaluating the cost-effectiveness of smoking cessation programs. In this regard, implementing our individualized support program mainly in smokers who were willing to quit but were severely dependent on nicotine may be have been less cost-effective than anticipated.

Although we consider that our study offers preliminary information on the cost-effectiveness of individualized smoking cessation support at a worksite in Japan over a relatively long-term period, several limitations in the study should be acknowledged. First, the design was a case study and not a randomized controlled trial. Some, but not all, participants used nicotine patches, and to some extent they elected whether they would use them on their own volition. Despite the marked influence nicotine patches are known to have on costs our analysis did not take into account whether the use of nicotine patches was due to the participants’ own volition or was at the physician’s discretion. This study required the users of nicotine patches to pay for 30% of the costs, which may have influenced their choice to use the patches. Participants who did not want to spend this money may have refused the use of nicotine patches despite the recommendation of the physician. However, such a situation is usual in real practice settings. Second, we estimated the natural quit rate on the basis of the two-year point-prevalence of abstinence in non-participating smokers that included some dropouts. The readiness to quit may have varied between the participating and non-participating smokers, and therefore it remains unclear how many of the non-participating smokers attempted to quit smoking over the two-year period. In addition, the duration of smoking cessation in the non-participating quitters and whether they had stopped by themselves were both unknown. These limitations require us to use caution when interpreting our data. The true net cost-effectiveness of our support program should therefore be evaluated in a randomized controlled trial. Third, we relied on self-reporting to assess smoking cessation status in both participating and non-participating smokers. In principle, smoking cessation status should be verified objectively. Fourth, we used FTND to assess the levels of nicotine dependence, although there is some evidence to suggest that FTND is not sufficiently sensitive to determine the degree of this dependence [40], [41]. Fifth, our results may not be generalized to other worksites in Japan and other countries because the implementation, success rate, and cost-effectiveness of smoking cessation programs may be influenced by complexities of internal dynamics at a specific worksite. Finally, effectiveness in our study was defined as smoking cessation, an intermediate outcome, but we did not evaluate cost-effectiveness in terms of other important clinical and humanistic outcomes, such as improvements in life expectancy and quality of life. Further investigations are required to generate evidence on this topic.

In conclusion, our study demonstrated that worksites can provide effective individualized support for smoking cessation by taking advantage of easy accessibility. Nicotine dependence status may be an important determinant for the cost-effectiveness of smoking cessation programs.
